# Pancreatic cancer: failures and hopes—a review of new promising treatment approaches

**DOI:** 10.37349/etat.2025.1002299

**Published:** 2025-03-18

**Authors:** Vittore Cereda, Mario Rosario D’Andrea

**Affiliations:** IRCCS Istituto Romagnolo per lo Studio dei Tumori (IRST) “Dino Amadori”, Italy; Asl Roma 4, Hospital S. Paolo Civitavecchia, 00053 Civitavecchia, Italy

**Keywords:** Pancreatic cancer, immunotherapy, KRAS, tumor microenvironment

## Abstract

Pancreatic cancer is a challenging disease with limited treatment options and a high mortality rate. Just few therapy advances have been made in recent years. Tumor microenvironment, immunosuppressive features and mutational status represent important obstacles in the improvement of survival outcomes. Up to now, first-line therapy did achieve a median overall survival of less than 12 months and this discouraging data lead clinicians all over the world to focus their efforts on various fields of investigation: 1) sequential cycling of different systemic therapy in order to overcome mechanisms of resistance; 2) discovery of new predictive bio-markers, in order to target specific patient population; 3) combination treatment, in order to modulate the tumor microenvironment of pancreatic cancer; 4) new modalities of the delivery of drugs in order to pass the physical barrier of desmoplasia and tumor stroma. This review shows future directions of treatment strategies in advanced pancreatic cancer through a deep analysis of these recent macro areas of research.

## Introduction

Pancreatic cancer is one of the most lethal malignancies worldwide. The lack of symptoms leads to late diagnosis, with almost 50% of patients having a metastatic disease. The American Cancer Society estimated that the five-year survival rate for advanced pancreatic cancer remains 3%, while in the past 15 years the five-years survival rate of patients with all the different stages of pancreatic cancer (from stage I to stage IV) increased to 13% [1–3]. Reasons of these numbers rely on the crucial value of earlier detection in the improvement of pancreatic cancer survival. At the same time these rates document how far we are from controlling the disease when it has spread to other organs. Up to now, systemic chemotherapy continues to play a fundamental role to increase survival, relieve symptoms and ameliorate quality of life in metastatic pancreatic cancer patients. Over the past 15 years, unfortunately just few randomized clinical trials, investigating different combinations of agents, showed improved outcomes (Table 1) [4]. The first drug that demonstrated a little but significant improvement in overall survival (OS) was gemcitabine, with a median OS of 5.56 months [5]. After the ineffective attempt to associate gemcitabine to erlotinib in 2007 [6], finally a significant benefit was reached by a combination strategy with FOLFIRINOX in the PRODIGE4/ACCORD 11 (NCT00112658) trial, as compared with gemcitabine alone [7]. Despite a relevant increased toxicity, for the first time, this treatment achieved a median OS of 11.1 months, a median progression-free survival (mPFS) of 6.4 months and an objective response rate (ORR) of 31.6% in advanced pancreatic cancer patients.

**Table 1:** Outcomes in randomized clinical trials of metastatic pancreatic cancer (first-line treatment)

**Chemotherapy**	**Phase**	**Number of patients**	**mOS**	**mPFS**	**ORR**	**Ref.**
Gemcitabine vs. 5-fluorouracile	II/III	126	5.56 months vs. 4.41 months (*P* = 0.0025)	2.33 months vs. 0.92 months (*P* = 0.0002)	5.4% vs. 0% (nc)	[5]
Gemcitabine plus erlotinib vs. gemcitabine	III	569	6.24 months vs. 5.91 months (*P* = 0.038, HR: 0.82)	3.75 months vs. 3.55 months (*P* = 0.004, HR: 0.77)	8.6% vs. 8% (nc)	[6]
FOLFIRINOX vs. gemcitabine	II/III	342	11.1 months vs. 6.8 months (*P* < 0.001, HR: 0.57)	6.4 months vs. 3.3 months (*P* < 0.001, HR: 0.47)	31.6% vs. 9.4% (*P* < 0.001)	[7]
Gemcitabine plus nab-paclitaxel vs. gemcitabine	III	861	8.5 months vs. 6.7 months (*P* < 0.001, HR: 0.72)	5.5 months vs. 3.7 months (*P* < 0.001, HR: 0.69)	23% vs. 7% (*P* < 0.001)	[8]
Gemcitabine plus capecitabine vs. gemcitabine	III	533	7.1 months vs. 6.2 months (*P* = 0.08, HR: 0.86)	5.3 months vs. 3.8 months (*P* = 0.004, HR: 0.78)	19.1% vs. 12.4% (*P* = 0.034)	[9]
NALIRIFOX vs. gemcitabine plus nab-paclitaxel	III	770	11.1 months vs. 9.2 months (*P* = 0.036, HR: 0.83)	7.4 months vs. 5.6 months (*P* < 0.0001, HR: 0.69)	42% vs. 36% (*P* = 0.11)	[10]

mOS: median overall survival; mPFS: median progression-free survival; ORR: objective response rate; Ref.: references; nc: not calculated; HR: hazard ratio

At the same time the randomized phase III trial MPACT, conducted in 2013, evaluated the efficacy of the combination of gemcitabine with nab-paclitaxel vs. gemcitabine alone and showed a median OS of 8.5 months and a mPFS of 5.5 months. The most common reversible side effects were myelo-suppression, fatigue and peripheral neuropathy [8]. Discouraging results came from a phase III study, investigating the survival benefit of the combination strategy of gemcitabine and capecitabine [9]. Infact the GEM-CAP trial only showed a significant improvement in mPFS [5.3 months vs. 3.8 months in gemcitabine alone arm; hazard ratio (HR): 0.78], but not in median overall survival (mOS; 7.1 months vs. 6.2 months). Due to these data, gemcitabine plus nab-paclitaxel continues to be the preferred first line treatment in metastatic pancreatic cancer patients, even if in patients with good performance status FOLFIRINOX treatment showed a significant effect in terms of OS, progression-free survival (PFS) and ORR, exerting improved outcomes as compared to gemcitabine and nab-paclitaxel. Unfortunately, we do not have a comparison phase III trial between these two regimens, but recently a meta-analysis and a randomized phase III study gave us interesting information.

NAPOLI-3 trial compared nab-paclitaxel-gemcitabine against NALIRIFOX, a combination strategy, similar to FOLFIRINOX, with a lower dose of oxaliplatin (60 mg/m^2^ instead of 85 mg/m^2^) and with the use of liposomal irinotecan at 50 mg/m^2^. Median OS was superior with NALIRIFOX, as compared to nab-paclitaxel plus gemcitabine (11.1 months vs. 9.2 months; HR: 0.83). Furthermore, the study demonstrated a significant advantage for NALIRIFOX in terms of median PFS (7.4 months vs. 5.6 months; HR: 0.69) [10]. NAPOLI-3 was the first formally positive trial in metastatic setting in a decade. However, toxicity and cost of the new regimen limit its real clinical impact, as compared to the standard of care, represented by nab-paclitaxel plus gemcitabine.

Subsequently, a meta-analysis of 7 phase III studies on first-line treatment of metastatic pancreatic cancer was performed in order to compare OS, PFS, ORR and side effects of NALIRIFOX, FOLFIRINOX and nab-paclitaxel plus gemcitabine [11]. As expected, NALIRIFOX and FOLFIRINOX regimens lead to an improved efficacy, as compared to nab-paclitaxel plus gemcitabine (mOS: 11.1 months, 11.7 months and 10.4 months respectively; mPFS: 7.4 months, 7.3 months and 5.7 months respectively; ORR: 41.8%, 31.6% and 35% respectively). Notably, outcomes associated with nab-paclitaxel plus gemcitabine were not too much inferior to those of triplet chemotherapies, suggesting an increased ability to manage doublet side effects over the years.

A cautious patient selection, based on age, toxicity profile, performance status and prior adjuvant/neoadjuvant treatment, should be considered in pancreatic cancer first line. Furthermore, a biomarker-driven therapeutic decision should be encouraged in the near future, hoping that pre-clinical and clinical research will help us to choose the best therapy for every single patient.

New investigations on the value of *BRCA* mutations, high microsatellite instability/mismatch repair-deficient (MSI-H/dMMR) status, all *KRAS* mutations, including *KRAS G12C* mutations, and an accurate genomic profiling will be crucial for the improvement of clinical outcomes.

Regarding second-line therapies, several trials did not achieve favorable outcomes. In the past, CONKO-03 trial showed that OFF scheme, including oxaliplatin and 5-fluorouracile (5-FU) infusion, had a little but significant advantage in mOS, as compared to 5-FU alone (5.9 vs. 3.3) after a first-line gemcitabine therapy [12], but subsequently mFOLFOX-6, a more manageable chemo, including oxaliplatin, did not demonstrate the same benefit in the PANCREOX study in 2016 [13].

In the last 10 years the only phase III trial, showing a significant improvement in OS, was the NAPOLI-1. In 2023, a total of 117 patients received nanoliposomal irinotecan plus folinic acid and 5-FU and had a mOS of 6.1 months vs. 4.2 months in 149 patients assigned to folinic acid and 5-FU alone [14]. On the basis of these results, ASCO guidelines recommend that patients with advanced pancreatic cancer, which progress to a gemcitabine-based therapy, should receive 5-FU plus nanoliposomal irinotecan or standard irinotecan. However, up to know, different efforts have not substantially improved OS in patients with advanced pancreatic cancer, with a mOS less than 12 months.

Pre-clinical and clinical research are focusing on four pivotal fields: 1) sequential cycling of different cytotoxic agents alone or followed by target drugs and immunotherapies, in order to avoid mechanisms of resistance; 2) discovery of new predictive bio-markers, in order to select a specific patient population for the best treatment; 3) combination strategies, including radiotherapy/surgery associated with immunotherapies and/or target therapies in order to modulate the immunosuppressive tumor microenvironment (TME) of pancreatic cancer; 4) new modalities of the delivery of agents deeply into the neoplastic tissue of pancreas. The objective of this review is to accurately describe recent advances in all of these areas, with a strong attention to most promising approaches that could overcome mechanisms of resistance observed in conventional therapies. The use of new target agents, through the more accurate knowledge of genomic and molecular profiling, the rational synergistic combinations of different therapies, leading to the inevitable cancer cell death and the help of new ways to administered pharmaceuticals directly into the structure of pancreatic cancer are the focus of this report. Furthermore, a brief section discusses possible integration of these novel approaches with standard-of-care treatments in current clinical practice, with particular attention to ongoing clinical trials.

This is a narrative review, based on a literature search of the English PubMed/Medline indexed journals, using key search terms including “novel treatments” AND “immunotherapy” AND “target therapy” AND “delivery strategies” with “pancreatic cancer” AND “pancreatic ductal adenocarcinoma”, focusing on recent authoritative reviews and pre-clinical/clinical original articles from last ten years (up to January 2025).

## Fields of research

### Sequence cycling of treatments

Recently, some authors randomized 158 patients with metastatic pancreatic cancer to receive a sequential treatment, composed by nab-paclitaxel plus gemcitabine followed by mFOLFOX-6 vs. standard nab-paclitaxel plus gemcitabine, as first line therapy in a multi-institutional phase II trial (SEQUENCE trial) [15]. The experimental arm achieved a mOS of 13.2 months (the best first-line data ever), as compared to 9.7 months of the standard chemo, but side effects of the sequential regimen were significantly increased, with a higher incidence of grade 3/4 neutropenia and thrombocytopenia and 2 treatment-related deaths. A second phase II trial, called FOOTHPATH trial, investigating the efficacy of the sequential therapy NAPOLI/FOLFOX-6, failed to reach a significant advantage in OS, as compared to nab-paclitaxel plus gemcitabine (mOS: 11 months vs. 8.7 months respectively) [16]. These results are leading to a careful reconsideration of the single-agent dosage and time to exposure of existing cytotoxic regimens.

At the same time, several trials explored the feasibility of the maintenance therapy in terms of efficacy and toxicity. A phase II study analyzed the efficacy and the toxicity of 6 months of FOLFIRINOX (Arm A) vs. 4 months of FOLFIRINOX followed by leucovorin plus 5-FU maintenance therapy (Arm B) and showed that OS was comparable between the two arms (10.1 months vs. 11.1 months), and that, unexpectedly, severe neurotoxicity was higher in the maintenance group (10.2% vs. 19.8%), probably because of the reintroduction of oxaliplatin at the disease progression [17].

One of the most interesting and debated phase III trial of maintenance regimen was POLO trial. 154 metastatic pancreatic cancer patients with a germline *BRCA1/2* mutation and a stable or responding disease after a 4-month platinum-base chemotherapy, were randomized to receive a poly-ADP-ribose-polymerase inhibitor (PARPi), olaparib, as maintenance therapy or placebo. The study showed a significant greater PFS with olaparib, as compared to placebo group (7.4 months vs. 3.8 months, respectively, HR 0.53), but there wasn’t an OS benefit (19 months vs. 19.2 months, respectively) [18]. FDA and EMA did approve olaparib, as maintenance therapy in platinum-sensitive metastatic pancreatic cancer patients with *BRCA1/2* mutations. Subsequently, a phase II single-arm trial, RUCAPANC2, demonstrated that rucaparib, administered as maintenance therapy, in patients with advanced pancreatic patients, with germline or somatic BRCA or PALB2 mutations and sensitive to platinum-based treatment, is able to reach a mOS of 23.5 months [19]. Furthermore, other authors explored the hypothesis of the increase of PARPi efficacy, adding immunotherapy in BRCA mutated patients. We describe these attempts (PARPVAX and POLAR trials) in a different section.

### Predictive biomarkers in pancreatic cancer

#### Molecular profiling

Different studies performed genomic and molecular profiling of patients with pancreatic cancer. A mean of 50–60 somatic mutations for patient were observed, revealing wide intratumoral heterogeneity. *KRAS*, cyclin-dependent kinase inhibitor 2A (*CDKN2A*), tumor protein 53 (*TP53*), and *SMAD* family member 4 (*SMAD4*) have been found to be the most frequent mutated targets [20]. In recent years, multiple reports have shown that *BRCA1*, *BRCA2* and *PALB2* genes, which are known as “the core homologous recombination (HR) genes”, present germline mutations in up to 5% of patients with pancreatic cancer. Beyond these alterations, germline and somatic *ATM*, *ATR* and *RAD51c* mutations have been demonstrated to increase susceptibility to pancreatic adenocarcinoma. Park et al. [21] showed that 71% of core HR gene alterations in pancreatic cancer are biallelic (functional). However, analysis of single HR gene mutations is insufficient to identify all pancreatic cancer patients with HR deficiency and a specific nucleotide variant substitution signature, COSMIC signature 3, or several genomic algorithms, including classifier of homologous recombination deficiency (CHORD) and HRDetect, have been developed to amplify the population with a “targetable” HR deficiency [22].

Over the years, several classifications were identified because of genomic and molecular analysis of pancreatic cancer. Regarding various genomic and molecular profiling, performed over the years, in 2015 Moffitt et al. [23] identified two tumor subtypes: classical and basal-like. Subsequently, the PURIST (Purity Independent Subtyping of Tumors) tool demonstrated that this tumor-intrinsic two-subtype schema was the most robust and replicable classification [24].

Data of the Comprehensive Molecular Characterization of Advanced Pancreatic Ductal Adenocarcinomas for Better Treatment Selection (COMPASS) trial, a recent prospective cohort study, assessed the response of first-line chemotherapy with mFOLFIRINOX or nab-palitacel plus gemcitabine based on these subtypes [25]. It was demonstrated that patients with a classical-subtype pancreatic cancer treated with mFOLFIRINOX had a favorable mPFS of 8.5 months, as compared to patients with a basal-like subtype (2.7 months). Increased expression of GATA6 was significantly associated with a classical subtype, suggesting a possible use of this classification to predict response to standard of care treatments [25]. Now, we are attending the results of PASS-01 trial (NCT04469556), a randomized phase II trial, which is evaluating outcomes of mFOLFIRINOX and Nab paclitaxel plus gemcitabine on the basis of GATA6 expression.

#### KRAS

*KRAS* mutations occur in 90–92% of pancreatic ductal adenocarcinoma (PDAC). The most common mutations are present in codon 12, codon 13 and 61. *KRAS G12D* mutations are 40% and are associated with worse outcomes. *KRAS G12V* (29%), *G12R* (15%), *G12C* (1%) are less frequent. In pancreatic cancer, *KRAS* mutations are often accompanied with inactivation of *TP53*, *CDKN2A* and *SMAD4*. It has been demonstrated that pancreatic cancer cells harbor early *KRAS* mutations and this is associated with an immunosuppressive environment in animal models [26].

For this reason, *KRAS* was considered the most important target in which to invest for PDAC therapy. Until now there have been several attempts to make *KRAS* a therapeutic target, most of them unsuccessful [27].

Oncogenic activation of *KRAS* is affected by both the specific mutation and the existence of extracellular stimuli. Indeed, various studies have demonstrated that mutated *KRAS* is subjected to rapid nucleotide cycling and its activation is sustained by enhanced upstream and downstream signaling [28]. It should also be emphasized that biochemical profiling of different mutant forms of *KRAS* has revealed intrinsic differences in both kinetics and biochemical properties between the same mutations [29].

Due to the initial difficulties in directly targeting *KRAS*, researchers have studied various indirect targeting methods, for example using Farnesyl transferase inhibitors (FTIs), *RAF* inhibitors (e.g., vemurafenib) and mTOR inhibitors (e.g., everolimus), all of which failed to achieve any beneficial effect [30].

However, the *KRAS G12C* mutation has recently been successfully targeted, using mutant-specific covalent inhibitors that bind a novel allosteric pocket [31], which led to the FDA approval of the *KRAS G12C*-specific inhibitor AMG-510 (sotorasib).

A phase 1–2 study assessed the clinical outcomes of sotorasib treatment in 38 patients with pancreatic cancer, harboring a *KRAS p.G12C* mutation and receiving at least one prior treatment. Authors reported that 8/38 patients had an objective response [21%; 95% confidence interval (CI), 10 to 37], the mPFS was 4.0 months (95% CI, 2.8 to 5.6), and the mOS was 6.9 months (95% CI, 5.0 to 9.1) [31].

An additional *KRAS G12C* inhibitor, adagrasib (MRTX849), has recently emerged. In a phase 1/2 multicohort study (KRYSTAL-1, NCT03785249), adagrasib was administered at a dosage of 600 mg BID to patients with advanced solid tumors carrying a *KRAS G12C* mutation. Among 21 patients with PDAC, ORR was 33.3% (7/21); DCR was 81.0% (17/21); median PFS was 5.4 months (95% CI 3.9–8.2); and median OS was 8.0 months (95% CI 5.2–11.8) [32].

Other new covalent *KRAS G12C* inhibitors are recently demonstrating higher activity with an ORR > 40% in pancreatic cancer [33–35].

Recently, there are in development multiple drugs targeting *KRAS G12D* (40% of KRAS mutations). MRTX1133 is a selective, non-covalent G12D inhibitor with promising results achieved in mouse models, obtaining > 30% of tumor regression in 8 of 11 pancreatic cancer models [36]. Moreover, pre-clinical and clinical investigations on different pan-*RAS* inhibitors, such as RMC-6236 or BI-2852, are in progress [37, 38]. In particular, RMC-6236 is an oral, direct RAS(ON) multi-selective inhibitor that suppresses RAS signaling by blocking the interaction of RAS(ON) with its downstream effectors. A phase 1/1b study (RMC-6236-001), a multicenter, open-label, dose-escalation and dose-expansion trial was designed to evaluate RMC-6236 as monotherapy in patients with advanced solid tumors, harboring *RAS* mutations or wild-type *RAS* [37]. A total of 127 patients with metastatic pancreatic cancer were treated at the doses from 160 mg to 300 mg once daily. The most common reported grade > 3 adverse events were rash (8%), stomatitis (3%) and diarrhea (2%) and dose modifications, due to toxicities, were reported in 35% of patients with no treatment discontinuations. Patients with metastatic pancreatic cancer, harboring a *KRAS G12X* mutation in the second-line setting, achieved a mPFS of 8.5 months (95% CI, 5.3–11.7) and a mOS of 14.5 months (95% CI, 8.8–NE). Lower mPFS of 7.6 months and mOS of 14.5 months were reported in patients harboring any RAS mutation. The OR rate for patients, harboring *KRAS G12X* mutations, was 29% in the second line group and 22% in the third line and beyond [37]. A phase III registrational study, RASolute 302 is ongoing (NCT06625320).

#### BRCA

It has been demonstrated that the risk of pancreatic cancer is increased in patients with loss-of-function *BRCA1* and *BRCA2* mutations. The prevalence of these mutations among all patients with a diagnosis of pancreatic cancer ranges from 4% to 8%, while in the pivotal phase III study POLO the percentage of metastatic pancreatic cancer patients was approximately 6% [39].

The concept of BRCAness was introduced to describe the cellular deficiencies that phenocopy those occurring in BRCA1- and BRCA2-deficient cancer cells. One such characteristic is the hypersensitivity of BRCA1- and BRCA2-deficient cells to PARPis. Indeed, initially, it has been showed that just both BRCA1 and BRCA2 deficiencies were synthetic lethal with PARPi genetic inactivation, but through the help of several reports, it was clear that, beyond *BRCA1* and *BRCA2* mutation “per se”, HR deficiency, including *PALB2*, *RAD51*, replication protein A1 (*RPA1*), *ATR*, *ATM*, *CHK1*, *NSB*, *WEE1* alterations, is a key determinant of sensitivity to PARP inhibition, amplifying the previous concept of BRCAness [40].

Since 2015, several studies did show efficacy of PARPis in patients with metastatic pancreatic cancer harboring germline BRCA1 and 2 mutations [18, 19, 41] and also an increased response to platinum-based therapy [21, 42]. POLO trial and RUCAPANC2 trial [18, 19] did demonstrate the activity of PARPis (olaparib and rucaparib, respectively) as maintenance in pancreatic cancer patients with alteration of the homologous recombination repair pathway, even if it has not been observed an OS advantage, raising doubts about the real benefit of these drugs among scientific community. Other confounding results came from POLO trial: 40% of patients with BRCA mutations progressed during first-line platinum-based therapy, a high percentage, considering the expected sensitivity to platinum in this subpopulation [18]. Moreover, a phase II study, conducted in 2020, analyzed outcomes of pancreatic cancer patients with *BRCA1/2* or *PALB2* mutations, treated with cisplatin-gemcitabine + veliparib and did demonstrate a favorable ORR (65%) with chemotherapy but not a clear benefit with the addition of PARPi (74% of ORR, 10.1 months of PFS vs. 9.7 months, 15.5 months of OS vs. 16.4 months) [43]. Recently, several authors showed different possible mechanisms of resistance to both platinum chemotherapy and PARPis in HR-deficient patients, including HR genes reversion mutations, reversing epigenetic modifications or ATR activation through replication fork stabilization [44]. PARP and ATR inhibitors were combined in clinical trials with upsetting outcomes due to high myelosuppression. Clinical trials are currently testing multiple dosages of this combination [22]. Moreover, mechanistic studies have revealed that inhibitors of DNA damage repair, such as PARP and ATR inhibitors, activate the cyclic GMP-AMP synthase (cGAS)/stimulator of interferon genes (STING) pathway, resulting in the secretion of type I interferons and promoting CD8^+^ T cell priming and migration [22, 45]. Based on these data, clinical research is focusing on the reinforcement of the efficacy of PARPis, using the combination with immunotherapy. We’ll describe this strategy in immunotherapy section.

#### ATM

Germline and somatic *ATM* mutations occur in 5–10% of patients with pancreatic cancer. ATM activates the HR pathway in response to double-strand DNA breaks, but PARP inhibition in patients with ATM mutations was disappointing [46]. It has been demonstrated that ATM, ATR and DNA-PK are three phosphatidylinositol 3-kinase-related protein kinases that can compensate for one another if one of the three proteins is deficient. In preclinical models, inhibition of DNA-PK and ATR are demonstrated to be synthetically lethal in ATM altered tumors [47].

Interestingly the BAY 1895344 ATR inhibitor showed a good clinical activity in pancreatic patients with *ATM* deficiency in a phase I trial [48]. Moreover, in pancreatic cell lines and in a murine model, the combination of gemcitabine with the AZD6738 ATR inhibitor have shown synergistic activity, leading to program new clinical studies in this field [49].

#### Replicative stress biomarkers

It has been well demonstrated that an uncontrolled cellular division in pancreatic cancer leads to high levels of replicative stress, which generates several single-strand DNAs, stimulating the ATR-CHK1-WEE1 pathway activation [50]. Firstly, ATR is activated by replication protein A (RPA) and propagates the DNA damage signal to CHK1 serine/threonine kinase, which slows cell cycle progression by activation of the G2/M checkpoint negative regulator WEE1. This mechanism allows the repair of DNA [50].

Inhibition of these three replicative stress biomarkers is currently under investigation. Poor outcomes and high levels of myelosuppression were observed using single agent inhibitors [51]. Interestingly, preclinical studies showed that gemcitabine inhibits the production of dNTPs, slowing the formation of replicative forks and increasing at the same time replicative stress, but the inhibition of ATR-CHK1-WEE1 pathway blocks the mechanism of compensation, ultimately leading to cell death [52]. Low dose gemcitabine was then combined with the SRA737 CHK1 inhibitor in a clinical trial [53]. The combination therapy was well tolerated with low myelotoxicity, but ORR was 10.8% [53]. Moreover, the WEE1 inhibitor adavosertib associated with gemcitabine showed clinical activity in HR-proficient pancreatic cancer [22, 54]. Indeed, adavosertib is able to inhibit G2/M cell cycle phase, where HR-proficient cells are more likely to be forced and preclinical models showed that pancreatic cells with acquired resistance to PARPis are highly sensitive to adavosertib [22]. In a phase I trial the combination of radiation with gemcitabine and adavosertib in advanced pancreatic cancer patients demonstrated a mPFS of 9.4 months and a mOS of 21.7 months [55].

#### MSI-H

Functional loss of mismatch repair (MMR) gene family (*MLH1*, *MSH2*, *MSH6* and *PSM2*) causes high microsatellite instability, leading to an increased risk of developing tumors and to a high probability of response to immunotherapy [56]. 1–2% of pancreatic cancer patients have this mutation [57].

Immunotherapy consists of the administration of monoclonal antibodies (mAbs), the immune checkpoint inhibitors (ICIs), which are able to modulate the patient’s immune system response, with exciting and long-lasting benefits in subgroups of patients, especially with gastrointestinal cancers. However, these benefits were not as consistent in pancreatic cancer [58].

Two phase II studies (KEYNOTE-016 and KEYNOTE-158) showed the efficacy of pembrolizumab, an anti-PD-1 antibody, in advanced solid tumors, harboring microsatellite instability/MMR gene deficiency with a ORR of 34% and a median PFS of 4.1 months [59, 60]. However, in these studies, pancreatic cancer patients had an RR of 18.2% and a median PFS of 2 months, suggesting a sort of resistance to immunotherapy in pancreas tumors, as compared to other cancers. The reasons of this behavior may be explained through deep knowledge of specific TME of pancreatic cancer, as described in Biologic rationale of combination strategies section.

#### Tumor mutational burden (TMB)

Tumor mutational burden (TMB) is a predictive biomarker of response to immunotherapy, which was investigated in different types of tumors [61]. TMB is the number of mutations per megabase (muts/Mb) in tumor cells and it has been documented that tumors with high TMB respond very well to immunotherapy. Unfortunately, just 1% of patients with pancreatic cancer have a high TMB and just 60% of them are also MSI-H, suggesting the actual controversy of using this phenotype as a valuable predictive biomarker [62].

#### Epigenetic aberrations

It has also been shown that in pancreatic cancer several epigenetic alterations cause changes in gene expression without affecting the DNA sequence. They are reversible, making them targets for tumor-directed therapies. The epigenetic changes occur through methylation of DNA, by histone modifications and through alteration of chromatin structures. Epigenetic aberrations seem to influence carcinogenesis, TME and immune cell function, leading to unexpected variations in treatment responses [63]. Several reports have shown that aberrant gene methylation is a frequent finding in pancreatic cancer [64]. Although monotherapies using epigenetic drugs, such as curcumin, a p300 histone acetyltransferase inhibitor and vorinostat, a hystone deacetylase (HDAC) inhibitor, are without benefit, targeting epigenetic modifications can alter susceptibility of pancreatic cancer toward standard of care chemotherapies [65, 66].

Table 2 shows results of the main clinical trials on agents targeting the most promising predictive pancreatic cancer biomarkers.

**Table 2:** Human studies on predictive biomarkers in pancreatic cancer

**Biomarkers**	**Target**	**Agent**	**Phase (number and characteristics of patients)**	**Outcomes**	**Ref.**
KRAS	*KRAS G12C* mutation	AMG-510 (sotorasib)	I/II (38 patients with at least a previous therapy)	ORR: 21%, mPFS: 4 months, mOS: 6.9 months	[31]
		MRTX849 (adagrasib)	I/II (21 patients with at least a previous therapy)	ORR: 33.3%, mPFS: 5.4 months, mOS: 8 months	[32]
BRCA	*BRCA1/2* germline mutation	Olaparib	III placebo-controlled (154 patients, as maintenance therapy with stable or responding disease after 4 months of platinum)	mPFS: 7.4 months vs. 3.8 months (HR: 0.53), mOS: 19 months vs. 19.2 months (HR: 0.83)	[18]
	*BRCA1/2* and *PALB2* germline and somatic mutation	Rucaparib	II single-arm (42 patients, as maintenance therapy with stable or responding disease after 4 months of platinum)	ORR: 41.6%, mPFS: 13.1 months, mOS: 23.5 months	[19]
MMR genes	MSI-high	Pembrolizumab	II single-arm (22 patients, after progression on standard therapies)	ORR: 18.2%, mPFS: 2.1 months, mOS: 4 months	[60]

MMR: mismatch repair; MSI-high: microsatellite instability high; mOS: median overall survival; ORR: objective response rate; Ref.: references; nc: not calculated; HR: hazard ratio; mPFS: median progression-free survival

### Combination strategies

#### Biologic rationale of combination strategies

The most important motivation for the resistance to systemic therapy and poor outcomes obtained from chemotherapy, immunotherapy or target agents appears to be the interactions between the TME [67, 68], pancreatic stem cells [69] and tumor cells [70]. Furthermore, hypoxia should not be underestimated as a factor of resistance to systemic treatments [71], because it causes a sort of desmoplastic reaction in the TME and leads to an increase in immune resistance and chemo-resistance. For this reason, although immunotherapy is a recent discovery, with stunning results obtained in melanoma and non-small cell lung cancer, it is not as promising to use for the treatment of PDAC, as we have seen.

To date, the most important obstacle to the effectiveness of immunotherapy (but also of some chemotherapies and other agents) in pancreatic cancer is an immunologically “cold” TME. It appears that the pancreatic tumor is protected by a structure, which works as a barrier against effective release of chemotherapeutic agents [72]. This structure is formed by a combination of malignant cells, fibroblasts of mesenchymal origin, blood vessels, pancreatic stellate cells and immune cells surrounded by extracellular matrix [73].

It has been demonstrated that carcinoma-associated fibroblasts (CAFs), often derived from pancreatic stellate cells, express fibroblast-activating protein (FAP), whose levels in the stroma are frequently associated with a worse prognosis for patients with pancreatic cancer [74]. Furthermore, FAP shows immunosuppressive properties in the TME and it has been demonstrated that depletion of FAP+ stromal cells increased the efficacy of anti-PD-L1 and anti-CTLA-4 treatments [75].

Both primary and metastatic pancreatic tumors are surrounded by a highly fibrotic stroma [76]. This stroma results from the production by CAFs of a variety of extracellular matrix proteins, as well as cytokines and vascular endothelial cells, all invaded by different immunogenic cells, such as lymphocytes, macrophages and mast cells. In orthotopic mouse models, co-inoculation of tumor cells and pancreatic stellate cells increased the size and metastatic potential of pancreatic tumors [77].

Many infiltrated immunogenic cells have been identified in the tumor stroma, such as tumor-associated macrophages (TAMs), myeloid-derived suppressor cells (MDSCs) and neutrophils [78]. At the same time, low levels of tumor-infiltrating lymphocytes (TILs) are imprisoned within the stroma as small clusters, leading to prevent them to directly interact with tumor cells [79]. Pancreatic cancer cells produce several molecules, such as CCL2 and GM-CSF, which are able to attract MDSCs and TAMs to the TME [80]. In particular, high levels of MDSCs have been correlated with a worse prognosis in PDAC patients with resected disease, while the presence of CD8^+^ and CD4^+^ T cells (effector T cells) appears to improve the prognosis of patients [80, 81].

Immunotherapy resistance in pancreatic cancer is also driven by its unique genetic landscape. It has been demonstrated that KRAS mutation, beyond its classic oncogenic role, can orchestrate a network of immunosuppression within the TME, directly preventing innate and adaptive antitumor immunity by amplifying autophagocytosis to modulate cell surface MHC class I (MHC-I) levels [82, 83] and by regulating the expression of CD47 and PD-L1 [84, 85].

Furthermore, this mutation coordinates a paracrine network aimed at the creation of a TME composed, as mentioned, of activated stromal cells and desmoplasia [86]. Indeed, KRAS mutation drives tumor-intrinsic expression of GM-CSF and CXCL1, consequently promoting MDSC TME invasion and the concurrent decrease of T cells [86].

**Figure 1:**
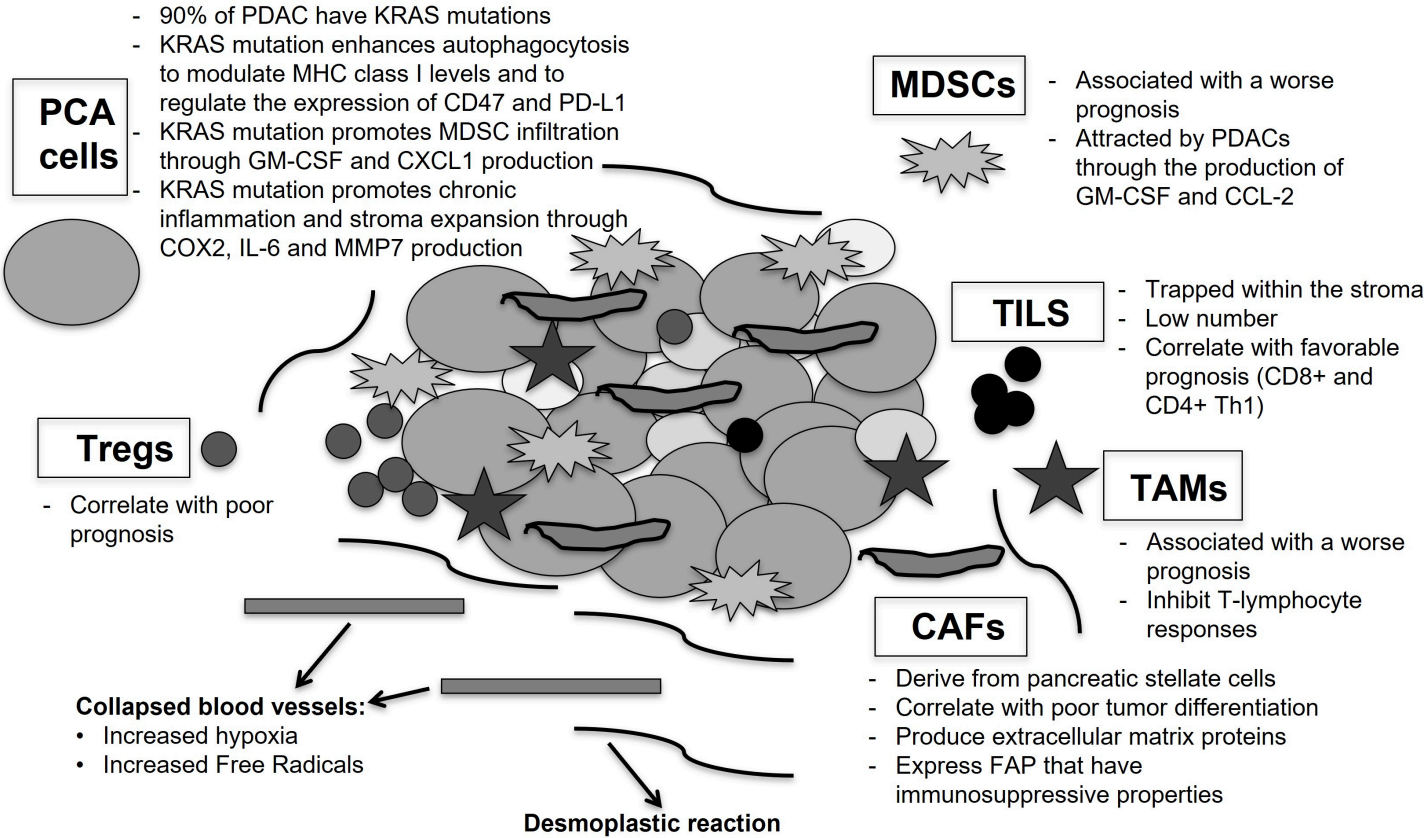
**Tumor microenvironment (TME) in pancreatic cancer and resistance to systemic therapy**. The figure shows the interplay between TME, tumor cells and immunogenic cells, which induces the increase of metastatic potential and immunosuppressive behavior of pancreatic cancer. Pancreatic cancer cells (PCA cells) with KRAS mutations lead the establishment of immunosuppression within the TME, modulating MHC class I levels and expression of CD47 and PD-L1; promoting myeloid-derived suppressor cells (MDSCs) infiltration, through the production of GM-CSF and CXCL1; increasing the levels of COX2, IL-6 and MMP7 with the expansion of fibro-inflammatory stroma (desmoplastic reaction). Carcinoma-associated fibroblasts (CAFs), derived from the activation of pancreatic stellate cells (PSCs), produce a variety of extracellular matrix proteins, forming a “barrier” against the release of chemotherapeutic agents and immunotherapies, and increase the levels of cytokines, inducing the migration of altered vascular endothelial cells and immunogenic cells with immunosuppressive properties. Infiltrated immunogenic cells are MDSCs; tumor-associated macrophages (TAMs) and Tregs, that are able to inhibit T-lymphocyte responses; tumor-infiltrating lymphocytes (TILs), that are presented in a smaller numbers and trapped within the TME. In this context the role of collapsed blood vessels should not be underestimated, because they increase hypoxia and the levels of free radicals, promoting cancer cells development and aggressiveness and helping to induce an immunosuppressive environment

mKRAS also activates Sonic Hedgehog signaling (SHH) and enhances the expression of COX2, IL-6, pSTAT3, and MMP7 favoring chronic inflammation and fibro-inflammatory stroma development [87]. Figure 1 shows the interplay between several “actors” in PDAC.

For these multiple biologic reasons and on the basis of poor results with single-agent immunotherapy or target therapy, recently several researchers are trying to achieve increased outcomes with combination of drugs with different mechanisms of action with or without the help of local approaches (double immunotherapy; immunotherapy + chemotherapy; immunotherapy + target agents; chemotherapy + target agents; radiotherapy/thermo-ablation/surgery + systemic therapy).

#### Dual immunotherapy

The first phase 2 trial, assessing the efficacy of ICI dual therapy, randomized 65 patients with mPDAC to be subjected to durvalumab (anti-PD-L1) monotherapy or durvalumab plus tremelimumab (anti-CTLA-4), after a first-line chemotherapy [88]. Outcomes obtained by the combination arm were an ORR of 3.1%, a mPFS of 1.5 months and a mOS of 3.1 months. Due to these discouraging clinical data, immunotherapy was then combined with current standard of cure chemotherapy regimens.

It has been shown that advanced pancreatic cancer with biallelic loss of BRCA1 and BRCA2 had a higher median tumor mutation burden than wild type tumors with the consequent possible higher sensitivity to ICIs [89]. Based on this concept, a retrospective single-institution case series involved 12 platinum-refractory metastatic pancreatic or biliary cancer with mutations in HR genes to be treated with 1 mg/kg of ipilimumab and 3 mg/kg of nivolumab every 3 weeks for 4 doses, followed by 480 mg of nivolumab alone, every 4 weeks [90]. Interestingly, 4/12 patients (30%) had a complete response, 1 had a partial response and 2 had stable disease. The OR rate was 42%, with a disease control rate of 58%. 3 out of 4 patients achieving a complete response remained free of disease after at least two years of treatment. Pre-treatment biopsies on 4 patients (2 with complete response and 2 with disease progression) documented significant differences in density of TILs and chemokines expression in favor of responders [90]. Despite small number of patients and few tumor samples analyzed, this work may serve as proof of concept for the use of dual immunotherapy in pancreatic patients with BRCAness phenoptype. However, recent a phase I/II (Chekmate 032) study on an unselected population with advanced pancreatic cancer previously treated with at least on chemotherapy regimen did not show significant objective response with this combination [91]. Future clinical trials need to be designed in specific metastatic pancreatic subpopulations.

#### Combination of chemotherapy plus immunotherapy

Numerous preclinical data indicate that chemotherapy-induced apoptosis can increase the immunogenicity of tumors through improved antigen presentation, T-cell reactivity, and T-cell tumor infiltration [92]. These studies supported the design of trials, investigating the activity of a combination of immunotherapy and chemotherapy in the hope of improving the antitumor immune effect. In the phase 1/2 PembroPlus study, it was reported that treatment-naive mPDAC patients, receiving the combination of pembrolizumab with nab-paclitaxel plus gemcitabine, had an ORR of 27% (3 of 11 patients with PR) [83], but the primary endpoint of > CR of 15% was not met [93]. In another phase 1 trial, Wainberg et al. [94] showed that the addition of nivolumab (anti-PD-1) to gemcitabine plus nab-paclitaxel in patients with untreated advanced PDAC achieved an ORR of 18%, a mPFS of 5.5 months, and a mOS of 9.9 months, suggesting that immunotherapy did not upgrade the outcomes of chemotherapy alone. In the exploratory analyses of this research, it was interestingly revealed that patients with longer mPFS (*P* = 0.03) had higher levels of peripheral CD8^+^ and CD4^+^ T cells during treatment, as compared to patients with poor outcomes. These findings, although exploratory, suggest that low levels of circulating CD8^+^ T cells may be a potential mechanism of resistance to ICI.

Subsequently, a randomized phase 2 trial by Renouf et al. [95] compared the efficacy of a first-line combination therapy of gemcitabine and nab-paclitaxel plus durvalumab (anti-PD-L1) and tremelimumab (anti-CTLA-4) with chemotherapy alone [85]. ORR (30.3% with ICI vs. 23.0% without ICI; *P* = 0.096), DCR (70.6% vs. 57.4%; *P* = 0.96), mPFS (5.5 months vs. 5.4 months; *P* = 0.91) and mOS (9.8 months vs. 8.8 months; *P* = 0.72) were not improved with the inclusion of immunotherapy in this chemotherapy regimen [95].

More recently, in another phase 2 study Morizane et al. [96] showed that 31 mPDAC patients, treated with first-line mFOLFIRINOX in combination with nivolumab, had an ORR of 32.3%, a mPFS of 7.39 months and a mOS of 13.4 months. These outcomes were again not statistically superior to those from patients treated with mFOLFIRINOX [7].

#### Combination of immunotherapy plus target agents

Some investigators are currently examining the activity of PARPi by increasing the antigen load, in order to favor the response to immunotherapy in patients with pancreatic cancer not selected by BRCA mutations.

In a recent phase Ib/II trial patients, who have not progressed to platinum-based first line therapy, were randomized to receive, niraparib plus nivolumab or niraparib plus ipilimumab, as maintenance [97]. The results were promising with a 6-month PFS of 20.6% (95% CI 8.3–32.9; *P* = 0.0002 vs. the null hypothesis of 44%) in the niraparib plus nivolumab group and 59.6% (44.3–74.9%; *P* = 0.045) in the niraparib plus ipilimumab group. However, it was reported a grade 3 or worse treatment-related toxicity in 22% of patients in nivolumab arm and in 50% of patients in ipilimumab arm. On the basis of these encouraging data, several studies are in progress to test this combination.

New findings from the phase 2 POLAR trial presented at ESMO congress 2024 showed favorable outcomes for patients with metastatic pancreatic cancer. The study enrolled 63 patients across three different cohorts: A) HRD patients with *BRCA1/2* or *PALB2* mutations; B) patients with non-core HRD mutations such as ATM; and C) patients with no HRD but with high response to platinum therapy. All patients received pembrolizumab plus olaparib. The most significant outcome came from cohort A with 64% of patients PFS-free at 6 months, a disease control rate of 90% and a mOS not reached at the moment [98].

#### Combination of systemic and local treatments

Recently, attempts have been made to integrate systemic therapies with local treatments. Percutaneous thermal ablations, such as radiofrequency ablation (RFA) or microwave ablation, irreversible electroporation (IRE) or stereotactic body radiation therapy (SBRT) are promising options for the management of patients with locally advanced disease. They are all feasible techniques for treating locally advanced pancreatic cancer (LAPC), with acceptable morbidity rates and a median OS of 23 months [99]. However, it has been documented that RFA can sometimes cause clinical issues when applied to pancreatic tumors due to the anatomical position of the organ located near fragile structures, such as the celiac trunk, the hepatic artery and the superior mesenteric vessels [99, 100]. In addition to the known “local” activities, there are numerous evidences that these local ablative therapies can both induce a systemic anti-tumor response with a potential abscopal effect [101, 102], and modify the TME turning what is in fact a “cold” into a “hot” tumor, increasing the infiltration of T cells and the production of pro-inflammatory cytokines [103].

Molecular mechanisms by which RT drive immune modulation are still not completely clear. We know that RT induces DNA damage through direct breakage of DNA as well as through generation of free radicals, leading to abscopal effect initiation [104, 105]. Type I interferons secreted by cancer cells, through activation of cGAS-STING pathway caused by RT-triggered DNA damage response, can facilitate dendritic cells (DCs) maturation, the increase of DCs co-stimulatory molecule expression and the DCs lymph-node migratory capacity. Damage-associated molecular patterns (DAMPs) are one of the most crucial molecular steps during the radiation-induced immunogenic cell death [106]. In addition to directly killing tumor cells, RT regulates tumor immune microenvironment. RT can stimulate the release of many-pro-inflammatory chemokines, including CXCL9, CXCL10, CXCL11 and CXCL16 from tumor cells and stromal cells, which promote the immune infiltration and increase the number of DCs, macrophages and T lymphocytes within TME [107]. Furthermore, it seems that RT reprogram the phenotype of TAMs from anti-inflammatory M2 like to CD8^+^- and CD4^+^-inducing M1 like ones [107]. Other mechanism by which RT increase the susceptibility of tumor cells to immune response-mediated cell death is the up-regulation of FAS expression, a member of the death receptor family [108].

Pre-clinical models, investigating RT’s mechanism of immune-stimulation, reported that RT can increase the antigen-processing and presentation pathways, particularly through the overexpression of MHC-I levels and may sometimes activate CD8^+^ T cells and cross-priming DCs [107]. Indeed, several reports showed that RT is able to reinvigorate exhausted intra-tumoral CD8^+^ T cells and/or to induce proliferation and differentiation of naive T cells [107]. It has been demonstrated that, besides is immune activating effect, RT may have also an immunosuppressive behavior, through induction of chronic type I interferons, upregulation of PD-L1 expression on tumor cell surface, enhancement of Tregs and lymphopenia [109]. Mainly, for these reasons, the ability of RT alone to act remotely is rare and limited to some clinical cases [105], but the combination of RT with modern immuno-stimulatory techniques has proven promising in various tumors, even in the metastatic setting [110].

Immunostimulatory mAbs, such as anti-CTLA-4 mAbs, anti-PD-1 mAbs, and anti-CD137 mAbs, have already been combined with RT in pre-clinical models, revealing a potent synergistic effect [111–113]. Results in humans have been promising in the sequential setting with either CTLA-4 antagonists [114] or PD-L1 inhibitors [115], especially for lung cancer patients. However, the optimal dose and timing of RT for the maximal abscopal effect is not fully understood. Recently some reports considered low-dose RT can better induce anti-tumor immune activation at the molecular level, but this combination warrants further in-depth research [116, 117].

The efficacy of ICI combined with RT in pancreatic cancer is suboptimal, primarily due to the unique and complex immunosuppressive TME. Mixed results have been reported using high or low RT doses in different investigations [118]. In some reports ablative RT is more effective than conventional graded RT for recruiting T cells [119]. In a recent phase II trial (NCT02704156), postoperative patients with locally recurrent pancreatic cancer received SBRT at doses ranging from 35 Gy to 40 Gy in combination with pembrolizumab with no improvement in OS [120]. In a phase II CheckPAC study patients with metastatic pancreatic cancer who were treated with nivolumab + ipilimumab + 15 Gy SBRT achieved an ORR of 37.2% compared with those treated with nivolumab alone (17%) [121]. Nonetheless, the majority of phase 1 and phase 2 clinical trials have failed to demonstrate efficacy in most patients [118]. The variability in the reported complication rates across studies may be due to the heterogeneity in treatment protocols used (dosage and timing) or may be related to the size of the tumors treated. A common complication is mild acute pancreatitis, while the more serious ones include severe acute pancreatitis, portal vein thrombosis, bile leak, perforations of the gastro-intestinal tract and pancreatic fistula [118, 122]. These adverse events are mainly caused by thermal injury.

Studies on efficacy of local ablative treatment in combination with immunotherapy are under way. RFA has a known immune effect in liver tumors [123] and its association with a CTLA-4 inhibitor achieved an encouraging benefit in a phase 1 study in hepatocellular carcinoma [124]. However, to date, feasibility of this “union” is limited by potential serious damage to adjacent tissues [125].

An interesting work by Giardino et al. [126] analyzed the influence of RFA on peripheral immune cells and demonstrated that CD4^+^, CD8^+^, and effector memory T cells levels increased from day 3 to day 30, while DCs increased later, suggesting that RFA may produce an effective adaptive immune response. At the same time, it was documented that circulating IL-6 decreased from day 3 to day 30, consistent with the supposed antitumor effect.

Alternatively, the mechanism of action of on IRE relies on short high-voltage electric pulses and for this reason it is considered a minimally invasive approach on surrounding tissues/organs. It has been reported that IRE modulates the peritumoral stroma and appears to lead to a transient significant decrease of Treg populations (CD4^+^CD25^+^, CD4^+^CD25^+^FoxP3^+^, and CD4^+^CD25^+^FoxP3^−^) in LAPC [127]. Recent literature confirms that IRE is able to cause a stronger abscopal effect than that of thermal ablation [128].

Indeed, this procedure could be a very favorable tool for the treatment of LAPC, limiting detrimental effect on the blood vessels and nerves, and modulating at the same time the immune response in unresectable PDAC [129, 130]. IRE carries some absolute contraindications, such as presence of metal implants, portal vein occlusion, epilepsy and myocardial contraction alterations. Several retrospective studies were performed, using IRE in pancreatic cancer patients. Complications depend on the team experience, the protocol used, the type of approach (open vs. percutaneous) and the size of the tumor, leading to percentage of adverse events ranging from 12% to more than 40% [131, 132]. A large retrospective study of 75 patients diagnosed with LAPC received percutaneous IRE post chemotherapy reported a 27-month mOS and a 15-months PFS. The procedure down-staged LAPC in 4 non-surgical candidates for surgical resection, mortality up to 30 days post treatment was 0% and the total amount of adverse events was 25% [133]. Subsequently, safety and efficacy of IRE were retrospectively investigated in 210 patients with LAPC and post-operative complications consisted in intra-abdominal hemorrhage, intra-abdominal infection and delayed gastric emptying (DGE) and a retrospective review noted that adverse events following IRE were similar to those of RFA or RT (i.e., sepsis, gastric leak, duodenal edema, porthal thrombosis, pseudoaneurysm bleeding, intra-abdominal abscess, biliary obstruction) [134]. An interesting retrospective study, comparing IRE to RFA after induction chemotherapy for patients with LAPC, showed that IRE had an increased survival 2-year benefit, as compared to RFA (53% vs. 27%), especially in tumors smaller than 4 cm [135].

These data paved the way for prospective trials. The PANFIRE II study, a multi-center, prospective, single-arm trial of 50 patients with locally recurrent pancreatic cancer or LAPC showed a mOS of 17 months from diagnosis. Interestingly, patients that received FOLFIRINOX induction chemotherapy, compared to patients that received gemcitabine or no therapy, did not achieved an improved survival [136]. In 2019, 54 patients with stage III/IV pancreatic cancer were enrolled to undergo IRE with or without chemotherapy [137]. No deaths relating to IRE procedure were reported, but common adverse events were ascites, pleural effusion, fever and abdominal pain with duodenal hemorrhage and portal vein thrombosis. mOS of patients with stage IV pancreatic cancer was 11.6 months in IRE group and 13.6 months in IRE + chemotherapy group. A post-hoc analysis on IRE-FOLFIRINOX group, compared to an historical FOLFIRINOX alone arm demonstrated and improved mOS (17 months vs. 12.4 months) for the combination treatment [137]. Recently, CROSSFIRE trial did not identify a difference in OS or incidence of adverse events between radiotherapy and percutaneous IRE after FOLFIRINOX [138]. mOS was 16.1 months in the radiotherapy group vs. 12.5 months in the IRE group (HR 1.39), while 25% in the IRE group had grades 3–5 adverse events (vs. 16% in the radiotherapy arm), including pancreatitis and cholangitis [138]. In the ASCO annual meeting in 2024, preliminary data of the DIRECT study on the safety of IRE after induction chemotherapy, when used for the ablation of stage III pancreatic cancer, showed that major adjunctive procedures were performed for more than 80% of the patients; the 90-day mortality was 5%; 25% of patients developed adverse events, including arterial hemorrhage, cardiac arrest and septic shock and one patient died for an intraperitoneal arterial hemorrhage [139]. High-frequency IRE is a new emerging technique, which may reduce the risk of muscle tetany and cardiac asynchrony, paving the way to other investigations [140].

Due to the immunomodulatory effect of IRE, combination of IRE with immunotherapy is an import area of research. To date, there are only two published trials with ICIs.

In 2020, O’Neil et al. [141] evaluated the peripheral levels of memory T cells after treatment with nivolumab (anti PD-1) associated with IRE and reported a significant enhancement of these immune cells from baseline to postoperative day 90, following the completion of immunotherapy (*P* = 0.009). No differences were found for CD4^+^ T cells, naive T cells, or central memory T cells.

In 2021, He et al. [142] showed a significant increase of the number of CD4^+^ (*P* = 0.038) and CD8^+^ (*P* = 0.024) T cells and a concurrent decrease of CD8^+^ Treg cells (*P* = 0.023) in patients treated with IRE plus toripalimab (anti PD-L1), as compared to those in the IRE-only arm.

Among ongoing clinical trials, PANFIRE III assesses the safety of IRE combined with IMO-2125 (toll-like receptor 9 ligand) and/or nivolumab in patients with metastatic pancreatic cancer (NCT04612530).

### New modalities of delivery of treatment into neoplastic tissue

As mentioned before, pancreatic cancer produces a sort of physical “barrier” against drugs. Furthermore, its anatomical localization could prevent an easy passage of agents deep into the tumor tissue. Recent investigations are focusing on the improvement of drug delivery.

A nano-based drug delivery approach is able to directly target cancer tumor cells with improved agent cellular uptake. Advantages of applying nanomedicine to the therapy of pancreatic cancer are controlled and sustained release of drugs, lower systemic toxicity, reduced number of administration and overcoming of tumor barriers with higher penetration in TME [143]. Currently developed materials are categorized in polymeric nanoparticles (including natural and synthetic ones), liposomes, micelles, exosomes, natural membrane-coated nanoparticles (including leukocyte-like carriers), viruses and inorganic nanoparticles [144]. Combination of different strategies of delivery systems may be the key to overcome issues related to the single delivery formulation.

On of the most extensive studied molecule is hyaluronic acid (HA) that acts as a ligand for several cell surface receptors, such as CD44 and CD168 receptors, which are overexpressed in human tumors [145]. Its use as single agent did not achieve successful outcomes, because of poor accumulation in solid tumors, due to the superficial penetration depth, low cellular uptake and non-specific drug release [145, 146]. The combination of HA and other molecules included in the drug delivery system not only maintains the ability of HA to target cancer cells, but also provides the system with the ability to deliver drugs through multiple interactions with TME of solid tumors. One of the main applications employs coating of nano- and microtechnologies with HA for the delivery of anticancer drugs and nucleic acids, such as DNA or small interfering RNA (siRNA) [146–148]. Multifunctional HA-based liposomal or polymeric nanoparticles drug delivery systems are currently designed to respond to specific stimuli, such as change in pH, temperature or enzyme activity. Dual targeting, releasing NO for collagen degradation or HA modifications are under investigation [148–152].

Regarding functionalization of nano-vehicles for drug release, the overexpression of folate receptors in tumors is a well-known occurrence and serves as the foundation for targeted cancer treatments that uses nanoparticles [153, 154]. By attaching specifically to folate receptors, folate-anchoring can improve the precise delivery of drug-loaded chitosan (a bio-polymer) nanoparticles and a folate-chitosan-gemcitabine compound is currently under investigation [155]. Evidence levels for the safety of drug transporters need to be rigorously established in the pre-clinical setting.

The efficacy of lipid-based nanoparticles depends on the enhanced permeability and retention effect (EPR), which is common for tumors [156]. EPR minimizes the dissemination of nanoparticles with a selective concentration within the malignant tissue. Lipid-based nanoparticles provide several benefits, including easy formulation, self-assembly, biocompatibility, capacity of large payloads and various physiochemical features [157].

Recent efficacious activity was achieved by a pegylated liposomal irinotecan (NALIRI), approved by the FDA back in 2015 in order to be used as second-line treatment for metastatic pancreatic cancer [158]. In pre-clinical investigations, liposomal irinotecan has demonstrated significant longer tumor SN-38 duration over 100 h than the 40 h of un-encapsulated irinotecan [159]. Numerous attempts in improving delivery of drugs with nanomedicine are under careful analysis [160–163].

On the basis of these previous findings, convincing results were seen in 2 phase III studies [10, 14]. Regarding drug delivery systems, most peptides can be coupled to drugs via linkers to form peptide-drug conjugates ad act active pro-drugs, because they have high affinity, low immunogenicity and adjustable molecular size [164]. CEND-1 is a promising example of peptide-based delivery compound. It is a novel cyclic peptide that targets aV integrins and neuropilin-1 and was administered in association with gemcitabine and nab-paclitaxel in an Australian phase I study. This peptide demonstrated to increase the delivery of this chemotherapy and its co-administration showed a 59% (17/29) of objective response (1 complete response and 16 partial responses) with a mOS of 13.2 months [165]. Future randomized trials are awaited.

Chimeric antigen receptor (CAR) T-cell therapy is a promising approach especially against tumors resistant to standard therapies, such as pancreatic cancer. Indeed, a recent field of research, regarding new modalities of drug delivery, is focused on CAR-T cell-based therapy. CARs are synthetic trans-membrane receptors expressed on genetically modified T lymphocytes, which are able to recognize specific surface antigens on cancer cells and kill them [166]. Antigen selection remains a significant challenge for CAR-T strategies targeting pancreatic cancer. Most efforts have focused on tumor-associated-antigens, which often exhibit variable and heterogeneous expression among tumor cells, posing a high risk of on-target, off-tumor toxicity. Antigens currently under investigation for CAR-T therapy in pancreatic cancer are CEA, MUC-1, HER-2, EGFR, CD133 and claudin 18.2 [167, 168].

Since now, safety and dosing evaluations were performed in patients with metastatic pancreatic cancer. Several phase I clinical trials also monitored the efficacy of the treatment with partial responses in few patients [169–172]. In particular, NCT02159716, a phase I study, investigated lentiviral-transduced CAR T-cells targeting mesothelin in 5 patients with metastatic pancreatic cancer. 3 patients showed no response and two had stable disease for just 2–3 months. The therapy was generally well tolerated, except for one instance of grade 4 toxicity (sepsis) [173]. An investigation with fully human anti-mesothelin CAR is underway (NCT03054298, NCT03323944). Poor outcomes were achieved with a phase I trial (NCT01869166), exploring EGFR CAR-T cells in 14 metastatic pancreatic cancer patients after chemotherapy regimen consisting in nab-paclitaxel plus cyclophosphamide [170]. All patients had grade > 3 reversible side effects, including fever, fatigue, nausea, vomiting, pleural effusions and pulmonary interstitial exudation. 4 individuals manifested a partial response and 8/14 had stable disease for 2–4 months. The mPFS was 3 months following the first EGFR CAR-T cycle and the mOS was 4.9 months [170]. At the same time, a HER2 CAR-T cells treatment administered to 2 pancreatic cancer patients, after a nab-paclitaxel-based chemotherapy, did show grade 3 toxicities, including febrile syndrome and upper gastrointestinal hemorrhage [171]. Two phase I trials investigated the utility of autologous CT041 CAR-T cells against claudin 18.2 and patients experienced grade 1 or 2 cytokine release syndrome and hematological toxicities with poor efficacy outcomes [172, 174]. In a phase I clinical study evaluating CD133 CAR-T cells (NCT02541370), 7 patients with stage IV pancreatic adenocarcinoma had grade 3 hematological adverse events; 2 showed no response, 3 maintained stable disease for 3–10 months and 2 exhibited partial remission for just 2–4 months [175]. In general, partial responses or stable diseases were achieved only in a small fraction of patients. In all of these preclinical and clinical studies it has been emphasized that CAR-T cell therapy success in pancreatic cancer treatment is hindered by TME. Intra-tumoral hypoxia, immunosuppressive cytokine profile and stromal desmoplasia decrease CAR-T cells extravasation, infiltration and persistence in the TME [168, 176]. Other limitations that hamper the efficacy of CAR-T cells in pancreatic cancer are heterogeneous antigen expression and cell-mediated toxicities. Different strategies to overcome these challenges are under investigation [168, 176]. Local (intra-tumoral) administration or the generation of CAR-T cells expressing enzymes, such as heparanase [177], and chemokine receptors (CXCR1 and CXCR2) [178] may overcome physical limitations. Dual-targeting CAR-T cells (e.g., anti-mesothelin plus anti-CD19) was tested to overcome antigen escape down-regulation [179]. Moreover, in order to fight against immunosuppressive microenvironment, generation of CAR-T cells expressing pro-inflammatory cytokines (e.g., IL-12 and IL-27) or combination with chemotherapy and immunotherapy are ongoing [168, 180]. Significant challenges in CAR-T cell therapy are cytokine release syndrome, immune effector cell-associated neurotoxicity syndrome and on-target, off-tumor toxicity [168]. CAR-T cells with a decreased affinity on the antigen-binding domain and inhibitory CAR-T cells may be potential strategies to control adverse events [168, 176, 181]. The use of next-generation CAR-T therapies, harboring multiple genetic modifications could be the way for the successful application of this strategy against pancreatic cancer [168, 176].

#### Current challenges and perspectives in nanomedicine approach

Nanomedicine in pancreatic cancer has yet to reach its full potential. Reasons of the challenges in overcoming the barriers posed by this malignancy are still not clear. Some reports showed that dynamicity of leakiness of tumor vessels and the presence of thrombi cause heterogeneity in the EPR effect in pancreatic cancer, leading to alteration in compound extravasation. However, stromal barriers within TME further attrite the efficacy of nanoparticles even after extravasation [143, 182]. Prominent fibrosis is a histopathological hallmark of pancreatic cancer and the median thickness of the fibrotic tissue is around 10–30 μm. The key cellular mediators of fibrosis are CAFs, whose heterogeneity stems from their diverse origins. Indeed, heterogeneity and dynamicity of ECM during pancreatic tumor progression hampered the efficacy of the nano-based drug delivery system, temporarily altering penetration and distribution of agents [143, 183].

One of the most studies pathways during formation of desmoplasia in pancreatic tumors is SHH signaling. Tumor cell-secreted SHH activates paracrine signaling in stromal cells and promotes fibrotic changes such as myofibroblastic differentiation and increased ECM deposition [72, 73]. Stromal ablation, through SHH inhibition may improve nanomedicine delivery, as documented in in-vivo models [184]. Another key regulator of collagen deposition is TGF-β, through activation of SMAD2/3 downstream in pancreatic stellate cells [73]. The direct targeting of TGF-β is considered clinically challenging due to its myriad functions across multiple organ system [185]. Other therapeutic drugs, used in fibrotic conditions, such as pirfenidone or tranilast, have been tested with unsuccessful outcomes. These stromal ablation strategies did not significantly increase penetration a distribution of nanoparticles, because they mainly focus on fibroblasts [186, 187]. It has been demonstrated that complex interplay of fibroblasts with other various cell types is the main obstacle to the efficacious delivery of therapeutics. The failure of stromal ablation in clinical practice reveals the tumor-suppressive role of desmoplasia in pancreatic cancer, leading to strategies that reprogram the stroma instead to eradicate it. Targeting fibroblast abnormalities and metabolism is under investigation [186].

ECM deregulation in the fibrotic TME leads to tumor stiffening, high mechanical stress and high interstitial fluid pressure, limiting the delivery of nanomedicine [182, 186]. Enzymatic degradation of hyaluronan and collagens and targeting ECM remodeling and signaling may overcome these obstacles. The optimization of nanomedicine design will likely also be important to defeat the fibrotic barrier in pancreatic cancer. It has been proved that less than 1% of injected nanomedicines accumulates in tumors and only 0.001% interacts with cancer cells [186]. The non-payload part of formulations is not inert, affecting the efficiency of delivery. Size of nanoparticles is crucial: small ones permit their penetration in fibrotic stroma but at the same time pose an engineering challenge when the payload is of large molecular weight [188]. In addition, modifications of ECM density and composition alter the ability of a nanoparticle with a certain size to penetrate it. An additional problem to solve is the efficiency of retention: smaller particles more smoothly penetrate but dot not easily remain within tissues. For this reason, engineering strategies to achieve size modulation in response to different stimuli are underway. Fibroblast and ECM active targeting within a nano-construct can facilitate the penetration of drugs, while at the same time the design of smart polymers incorporating moieties that confer responsivity to stimuli, such as change in pH and redox status can help to exert the highest therapeutic effect in specific areas of the tumor [182, 186].

An important issue in clinical translation is also represented by the nanoparticle-bio interface, which determines the safety and efficacy of nanomedicine. It has been showed that corona formation on the surface of nanoparticles affects their functionality, while the emergence of immune responses against nanoparticles upon repeated exposures limits long-term efficacy and may influence patient safety [182]. Even if our understanding of the mechanisms driving fibrosis in pancreatic cancer is advancing, improving penetration and distribution of drugs deeply the into tumor core has experienced numerous disappointments. The design of new generation constructs, able to overcome the multiple obstacles posed by pancreatic cancer TME, is key for cancer nanomedicine to be a sustainable strategy in the clinic for pancreatic cancer patients [186].

## Integration of novel therapies with standard-of-care treatments in advanced pancreatic cancer

The increasing landscape of clinical trials dedicated to advanced pancreatic cancer reflects a concerted effort within the scientific and medical communities to address the pressing need for more effective treatment options in clinical practice [189]. Among more than 700 active studies, just 25 trials are in phase III with a substantial focus on more advanced stage of clinical testing. 18/25 ongoing phase III trials are investigating novel combinations with established drugs like gemcitabine, fluouracil and paclitaxel, aiming to enhance therapeutic efficacy and overcome resistance mechanisms.

Some phase III trials investigate the combination of standard-of-care treatments with novel agents targeting the components of stroma, such as pamrevlumab, a new fully human mAb that binds to connective tissue growth factor (cTGF) (NCT04229004) or a PEGylated recombinant human hyaluronidase in patients with advanced pancreatic cancer (NCT02715804). Targeting fibrosis in pancreatic cancer is crucial due to its significant impact on treatment efficacy and there are numerous early phase trials, testing new drugs that can modify TME. Among them, the current randomized phase II trial (NCT03727880) evaluates the use of pembrolizumab with a FAK inhibitor, defactinib, as sequential neoadjuvant and adjuvant therapy in patients with high-risk resectable pancreatic cancer.

A large amount of trials are focusing on immunotherapies in combination with several chemotherapeutics or novel agents. The majority of these investigate checkpoint inhibitors targeting the PD-1/PD-L1 or CTLA-4 axis in a very advanced stage of disease. The clinical benefit of ICIs in pancreatic cancer has been largely restricted to a small subset of patients characterized by dMMR, MSI-H and elevated TMB [72, 73]. Current consensus suggests that single-mechanisms immunotherapies are insufficient to fight pancreatic cancer. Ongoing clinical trials are focusing on combining ICIs with chemotherapy and/or radiotherapy to enhance anti-tumor responses in the majority of the patients. For instance, the CISPD-4 randomized phase II trial (NCT03983057) is investigating mFOLFIRINOX with or without anti-PD-1 antibody as neoadjuvant therapy in LAPC. Alternative immunotherapeutic interventions, such as cancer vaccines, TILs therapy, CAR cell therapy or TCR-engineered T-cell therapy, are at the early stage of development (phase I).

Research is also focusing on therapies targeting specific mutations in pancreatic cancer in combination with immunotherapy or chemotherapy. Based on the results of the POLO trial [18] and giving preclinical data showing that PARPis can activate immunostimulatory pathways [22, 44, 45], the SWOG0G2001 trial (NCT04548752) is assessing the combination of olaparib and pembrolizumab vs. olaparib alone as maintenance therapy in metastatic pancreatic cancer, with the primary objective of increasing mPFS. Clinical trials are also examining PARPis combined with FOLFIRI (NCT02498613). These combinations may increase the sensitivity to PARPis to a larger population than that with BRCA mutations. Moreover, conventional cancer treatments, such as radiotherapy and chemotherapy, often face resistance due to enhanced DDR mechanisms. Therefore, DDR inhibitors, such as ATM/ATR inhibitors, are being used in conjunction with these therapies to overcome such resistance. AZD0156 is under investigation in a phase I trial, either as monotherapy or in combination with chemotherapies and olaparib for advanced cancer patients, including pancreatic cancer patients (NCT02588105).

Although the development of KRAS inhibitors has transformed KRAS into a targetable protein, responses occur in only about 20–30% of pancreatic cancer patients and these responses are often partial and not durable [27]. Combining KRAS inhibitors with chemotherapy or co-targeting of EGFR may result in better antitumor effects; however, studies investigating this aspect are in pre-clinical setting [190].

Despite the promising research, there remains a significant challenge in identifying reliable biomarkers to predict therapeutic response and toxicity. Integrating biomarker-driven strategies into clinical practice is crucial for optimizing therapeutic efficacy and improving patient prognosis.

## Conclusions

Recently, significant advances have been made in deeply understanding the biology of the development of pancreatic cancer. Despite this improvement, up to now, there has not been a successful translation in clinical care for patients, with a median OS that remains poor for the majority of them. In our opinion focus on four pivotal themes may help to find the right “key” to finally increase outcomes of pancreatic cancer patients. Sequential and maintenance strategy; predictive genomic alterations and new target agents; modulation of TME and immunosuppressive features with combination therapy; and new modalities of drugs delivery are the promising fields, that need to be deeply explored. With the help of innovative clinical trial design, approaches, which encompass as many of these basics as possible, may represent the future of the treatment of pancreatic cancer.

## Abbreviations

5-FU: 5-fluorouracile
CAFs: carcinoma-associated fibroblasts
CAR: chimeric antigen receptor
CI: confidence interval
DCs: dendritic cells
EPR: enhanced permeability and retention effect
FAP: fibroblast-activating protein
HA: hyaluronic acid
HR: hazard ratio
ICIs: immune checkpoint inhibitors
IRE: irreversible electroporation
LAPC: locally advanced pancreatic cancer
mAbs: monoclonal antibodies
MDSCs: myeloid-derived suppressor cells
MMR: mismatch repair
mOS: median overall survival
mPFS: median progression-free survival
ORR: objective response rate
OS: overall survival
PARPi: poly-ADP-ribose-polymerase inhibitor
PDAC: pancreatic ductal adenocarcinoma
PFS: progression-free survival
RFA: radiofrequency ablation
SBRT: stereotactic body radiation therapy
SHH: Sonic Hedgehog signaling
SMAD4: SMAD family member 4
TAMs: tumor-associated macrophages
TILs: tumor-infiltrating lymphocytes
TMB: tumor mutational burden
TME: tumor microenvironment

## Declarations

### Copyright

© The Author(s) 2025.
